# Cardiac Troponins Metabolism: From Biochemical Mechanisms to Clinical Practice (Literature Review)

**DOI:** 10.3390/ijms222010928

**Published:** 2021-10-10

**Authors:** Aleksey M. Chaulin

**Affiliations:** 1 Department of Cardiology and Cardiovascular Surgery, Samara State Medical University, 443099 Samara, Russia; alekseymichailovich22976@gmail.com; Tel.: +7-(927)-770-25-87; 2 Department of Histology and Embryology, Samara State Medical University, 443099 Samara, Russia

**Keywords:** cardiac troponin, metabolism, mechanisms of troponin release, circulation, elimination of cardiac troponins, circadian, diagnostics

## Abstract

The metabolic processes of endo- and exogenous compounds play an important role in diagnosing and treating patients since many metabolites are laboratory biomarkers and/or targets for therapeutic agents. Cardiac troponins are one of the most critical biomarkers to diagnose cardiovascular diseases, including acute myocardial infarction. The study of troponin metabolism is of great interest as it opens up new possibilities for optimizing laboratory diagnostics. This article discusses in detail the key stages of the cardiac troponins metabolism, in particular the mechanisms of release from a healthy myocardium, mechanisms of circulation in the bloodstream, possible mechanisms of troponin penetration into other biological fluids (oral fluid, cerebrospinal fluid, pericardial and amniotic fluids), mechanisms of elimination of cardiac troponins from the blood, and daily changes in the levels of troponins in the blood. Considering these aspects of cardiac troponin metabolism, attention is focused on the potential value for clinical practice.

## 1. Introduction

Cardiac troponin isoforms (cTnI, cTnT, cTnC) are the most important regulatory proteins part of the troponin-tropomyosin complex localized on the actin (thin) myofilaments in cardiac myocytes. The protein molecule cTnI is an inhibitory subunit; it blocks the hydrolysis of adenosine triphosphate and the interaction of actin with myosin in the absence of calcium ions in the diastolic phase. The protein molecule cTnT is a tropomyosin binding subunit; it attaches two other troponin subunits to actin filaments. The protein molecule cTnC is a calcium-binding subunit; it binds calcium ions that enter the cytoplasm during the systolic phase [1,2]. The importance of cardiac troponins in the regulation of myocardial contractile function is demonstrated by the fact that small changes in the amino acid sequence of the protein molecules cTnI, cTnT, and cTnC are associated with significant and life-threatening violations of the contractile function of the heart’s muscular layer, known as hereditary cardiomyopathies [3,4]. The amino acid composition of cTnC is similar to the amino acid composition of troponin C located in the troponin complex in skeletal muscle fibers; therefore, this protein is not used as a laboratory biomarker for the diagnosis of acute myocardial infarction (AMI). At the same time, the amino acid composition of cTnI and cTnT protein molecules is unique, providing them with the necessary specificity for use in the diagnosis of AMI. However, clinicians should consider both the serum cTnI and cTnT levels and data from other diagnostic methods while diagnosing AMI [5,6,7]. This is because cTnI and cTnT molecules can be released from cardiomyocytes in various physiological (exercise, stress) and pathological conditions (sepsis, cardiac arrhythmias, myocarditis, pulmonary embolism, and others) are frequently accompanied by cardiac myocyte damage [2,8,9,10,11]. Thus, according to G. Lindner et al. (2014), AMI was diagnosed in only 175 of 1573 patients with elevated serum cTnT levels. In the vast majority of the patients examined (*n* = 1398), elevated cTnT concentrations were associated with other pathologies. The authors also noted that in 30% of patients, elevated cTnT levels were not associated with any of the causes of increased cardiac troponins described in the literature [12]. Thus, increased levels of cTnI and/or cTnT can reliably identify AMI only in the presence of clinical signs and/or symptoms indicating ischemia of cardiac muscle tissue. Furthermore, in modern diagnostic algorithms (0–1 h and 0–2 h) of early exclusion and/or confirmation of AMI, the dynamical change in the concentration of cardiac troponin levels within one or two hours after the patient’s admission to the department plays a critical role [6]. 

The diagnostic value of cTnI and cTnT depends on detection methods that constantly improve and change our understanding of biochemistry and broaden the possibilities for using cTnI and cTnT in clinical practice [13]. According to modern concepts, cardiac troponins in concentrations less than the established 99th percentile are considered to be normal myocardial metabolites. These concepts were formed due to a significant increase in the sensitivity (detecting ability) of laboratory methods. New generation methods, also called high- and ultrasensitive assays, have made it possible to identify cardiac troponins in blood and other biological fluids of all healthy patients [14,15,16,17]. Simultaneously, detected cardiac troponin concentrations (below the 99th percentile) in individuals without CVD have a strong predictive value [17], which can be used to identify people at higher risk of developing CVD for future implementation of a set of preventative measures to reduce this risk.

The levels of cardiac troponins, determined using modern immunological methods, depend on the metabolism of troponin proteins. The metabolism of troponins, like any endo- and exogenous compounds, can be conditionally divided into several stages: (1) the release of troponins from cardiomyocytes into the extracellular fluid and blood; (2) circulation in the bloodstream for a certain time; (3) degradation of troponins intra- and extracellularly into smaller fragments and their elimination; (4) troponin molecules can also be more actively released from myocardial cells at certain times of the day, contributing to the formation of circadian rhythms of cardiac troponins.

The relevance of studying the metabolism of troponin proteins is not only due to its great theoretical value but also has great practical value. First of all, this must be considered when interpreting elevated troponin levels in patients without established acute myocardial infarction (AMI), optimizing algorithms for AMI diagnosis, and improving the differential diagnosis of AMI [2,18,19,20,21]. Each method for determining cardiac troponins, among the many developed nowadays, gives different values, making it impossible to compare the same patient’s results obtained by test systems from different commercial kits [19,20,22]. This indicates that different immunoassays detect different molecules and/or fragments of cardiac troponin molecules [23]. In addition, different troponin molecules or their fragments are subject to the action of protease enzymes and therefore have different life spans in the blood (half-life) [24,25,26,27,28]. Furthermore, due to the increased activation of specific proteases, some troponin molecules can undergo increased destruction. Therefore, situations are possible in which diagnostic antibodies directed against these molecules may be ineffective, and the result of a laboratory study will be underestimated. The study of the half-life of circulating troponins and their fragments in the bloodstream is necessary for the development and subsequent use of more accurate immunoassays aimed at the most stable epitopes (antigenic determinants). 

Troponin molecules can also penetrate other human biological fluids (oral fluid, cerebrospinal fluid, pericardial and amniotic fluids), opening new questions and prospects for their use [20,28,29,30,31,32,33,34,35]. So, for example, the study of troponins in other biological fluids, particularly pericardial and cerebrospinal fluids, can be used in the forensic medical examination [31]; in amniotic fluid, to assess the condition of the fetus [33,34]; and in urine and oral fluid, for non-invasive diagnosis and monitoring of cardiovascular diseases [28,29,30,35,36,37].

## 2. Mechanisms of Troponin Release from Cardiomyocytes

With the development of highly sensitive assays and the subsequent detection of troponins in all of the healthy people, the attention of researchers is directed to the study of troponin release pathways from viable (intact) myocardial cells. The most studied mechanisms of troponin release from the myocardium of healthy individuals are the following: processes of regeneration and renewal of myocardial cells, apoptosis of cardiomyocytes, the release of troponin as a part of membrane vesicles, the release of fragments of proteolytic degradation of troponins, increased permeability of cardiomyocyte cell membranes, and small-scale (subclinical) myocardial necrosis [38,39]. It should be noted that some of the listed pathways can be enhanced in specific pathological processes and, accordingly, will be of great clinical importance.

### 2.1. Regeneration and Renewal of the Myocardium

Although the absence of a significant regenerative capacity of the myocardium is generally recognized, studies prove the myocardium has a small regenerative potential. Using the labeled ^14^C radioisotope integrated into the DNA of cardiomyocytes, evidence has been presented for the renewal of myocardial cells, the intensity of which decreases with age. Thus, in the age group up to 25 years old, around 1% of cardiomyocytes are divided every year, and the division activity steadily diminishes until it is just 0.45% by the age of 75. It was calculated that about half of the cardiomyocytes are replaced throughout a lifetime, indicating that the myocardium has a limited regeneration capacity. The calculation of the renewal rate of cardiomyocytes is based on assessing the rate of DNA synthesis produced by determining the rate of accumulation of the radioisotope in cardiomyocytes [40]. Presumably, the renewal process of cardiomyocytes is associated with the release of troponins into the bloodstream [41,42]. According to other data, the turnover of cardiomyocytes for mammals is 0.5–2% per year, and the frequency of cardiomyocyte renewal may be higher after injury than under normal conditions. Experimental assessment of myocardial regeneration in case of injury is complex due to the development of inflammation, the proliferation of stromal and vascular cells, and scar formation (sclerosis) [42,43]. It has also been shown that ischemic damage activates the internal regenerative potential of heart stem cells [43]. In addition, researchers Waring et al. (2014) and Rovira et al. (2018) in experimental studies on rats and zebrafish showed that regular exercise of moderate intensity promotes both hypertrophy of existing cardiomyocytes and activation and subsequent differentiation of myocardial stem cells with the formation of new myocytes [44,45].

### 2.2. The Role of Apoptosis in the Release of Troponins from Cardiomyocytes

The initiation of apoptosis is accompanied by an increase in the activity of caspase enzymes and intracellular proteinases, which leads to the cleavage of DNA and protein structures, with the relatively preserved cell membrane integrity [46]. The modern set of methods for detecting apoptosis is represented by electron microscopy, immunohistochemistry, flow cytofluorimetry, as well as the TUNEL method (Terminal deoxynucleotide Transferase—mediated dUTP—biotin Nick—End Labeling), which is considered the earliest (sensitive) and reliable criterion for apoptosis [47,48,49]. TUNEL analysis allows verification of the primary phenomenon of apoptosis—DNA decay caused by caspases. The principle of the method lies in the specific binding of terminal deoxynucleotidyl transferase with broken DNA strands, which can be detected by fluorescence microscopy [49].

Weil et al. (2017) experimented on pigs, simulating short-term (10-min) ischemia by balloon occlusion in the pool of the second diagonal branch of the left anterior descending artery. Complete occlusion was confirmed by contrast angiography. After reperfusion, the myocardium was removed post mortem for histological evaluation in some animals. Moreover, in another experiment on (live) animals, a series of measurements of troponin I were carried out after reperfusion using a moderately sensitive method (Life Diagnostics, West Chester, Pennsylvania) in blood serum samples obtained from the regional (anterior interventricular vein) and systemic (jugular) blood flow. Histological studies did not reveal signs of ischemic damage and necrosis. Apoptosis was confirmed by a sixfold increase in TUNEL-positive cardiomyocytes in the focus of short-term ischemia compared to the non-ischemic (control) area. The concentrations of cardiac troponin I were slightly increased after 10 min. After 30 min, they reached the values of the 99th percentile (38 ng/L) and continued to increase, reaching a peak at 24 h (1021 ± 574 ng/L). There was a very close and reliable correlation between the values of troponin I in the regional and systemic circulation. Thus, the authors noted that short-term ischemia does not lead to cardiomyonecrosis but leads to apoptosis of the cardiomyocytes accompanied by the release of troponins into the bloodstream [50]. Although it is worth noting that this study is limited to a time interval of 24 h, it is not known what would happen next with the concentration of troponins and with the state of cardiomyocytes. It is also worth noting that in actual clinical practice, such early detection of troponins by moderately sensitive techniques is difficult/practically impossible due to the wash-out phenomenon, in contrast to the ideally recreated reperfusion conditions in the experiment described above. 

Apoptosis of cardiomyocytes can also occur through mechanisms not associated with myocardial ischemia. Cheng et al. (1995) found an increase in programmed cell death in response to myocardial distension [51]. In this case, stretching of the myocardium can occur both in physiological conditions (for example, heavy physical exertion) and in several pathological conditions (for example, in heart failure and arterial hypertension), which possibly contributes to an increase in the level of troponins [52,53,54]. Singh et al. (2001) investigated the effect of enhanced neurohumoral stimulation on apoptosis processes and found that stimulation of beta1-adrenergic receptors induces apoptosis via activation of adenylyl cyclase [55], while stimulation of beta2-adrenergic receptors, on the contrary, has an antiapoptotic effect [56,57]. A decrease in the density (number) of adrenergic receptors is observed with age, which is more typical for beta2-adrenergic receptors [58]. Thus, there is reason to hypothesize a specific role of apoptosis in troponin elevation in heart failure, aging, prolonged and/or excessive exercise. An increase in troponins in these categories of patients can lead to overdiagnosis of AMI, especially when using highly sensitive test systems and in cases where clinicians rely on laboratory data alone to make a diagnosis. So, the work of Manjunath et al. (2018) acts as a confirmation. A young patient was admitted to the emergency department with chest discomfort, the troponin I concentration was increased 2.5-fold (0.123 ng/mL with a normal rate of <0.055 ng/mL). Doctors suspected myocardial infarction, additionally relying on unfavorable family history and the presence of hypercholesterolemia. However, ECG, Echo-CG, and coronary angiography did not reveal any signs of myocardial ischemia. From the anamnesis later, it turned out that the young man was actively involved in sports and ran several miles on the eve of admission, preparing for a marathon [59]. 

An experimental study in pigs showed that an increase in left ventricular preload leads to apoptosis and troponin I release without ischemia. The animals received phenylephrine (300 μg/min) for 1 h to increase the end-diastolic pressure. The basal release of troponin I was low (16 ± 20 ng/L), but 30 min after the increase in end-diastolic pressure, the concentration of troponin I increased above the 99th percentile, and after 1 h, it was 856 ± 956 ng/L (*p* = 0.01) and remained elevated after 24 h (1462 ± 1691 ng/L). Pathological analysis showed the presence of apoptosis of myocardial cells (31.3 ± 11.9 cardiomyocytes/cm^2^ versus 4.6 ± 3.7 cardiomyocytes/cm^2^; *p* < 0.01), which returned to normal after 24 h (6.2 ± 5.6 myocytes/cm^2^; *p* = 0.46) without signs of necrosis [60]. Thus, it was the process of apoptosis, not the necrosis of cardiomyocytes, responsible for the increase in the concentration of cardiac troponins [61,62].

### 2.3. Vesicular Troponin Transport

Cardiac troponins in the myocardium are represented by two fractions (pools): (1) associated (structural)—in the contractile apparatus; (2) cytoplasmic—freely located in the cytoplasm of cardiomyocytes and by volume accounts for approximately 3.5% of the total intracellular mass for troponin I and 7.0% for troponin T [63,64,65]. 

Animal hepatocytes and cardiomyocytes studies have revealed that during the early stages of ischemia, in the absence of necrosis, vesicles form on the surface of the cell membrane, containing cytoplasmic proteins, including troponins. The release of troponins occurs when these vesicles rupture on the surface of the cardiomyocyte. Schwartz et al. (1985), for the first time, studied the features of the formation of vesicles on the surface of the membranes of cultured cardiomyocytes using electron microscopy and noted a significant increase in the number of vesicles 30 min after ischemia, compared with the initial state [66]. 

This hypothesis is consistent with the concept of biphasic troponin release following irreversible damage. The initial phase is associated with the release of the cytoplasmic pool of troponins. In the case of rapid elimination of the causative factor (for example, ischemia, heavy physical exertion, psychoemotional stress)—reversible damage—the vesicles with their contents come back, and everything ends there. However, if the ischemia is more prolonged, for example, in myocardial infarction, then the second phase begins—on the surface of the plasmalemma of the cardiomyocyte, an avalanche-like formation of vesicles occurs with the destruction of the membrane; in parallel to this, there is slow lysis of the sarcomere, which includes a structural pool of troponins, and troponin circulation in the blood takes significantly longer [67].

Thus, it can be suggested that conditions such as prolonged/excessive physical activity, psychoemotional stress, ischemia, and increased load on the myocardium are also accompanied by the release of troponins through membrane vesicles.

### 2.4. The Release of Troponin Proteolytic Degradation Fragments from Cardiomyocytes

Myocardial tissue and the liver and kidneys are essential organs that ensure acid-base balance through lactate utilization. Lactic acid is formed in significant quantities by skeletal muscles and is delivered to the liver, where, under the action of lactate dehydrogenase (LDH), it is converted into pyruvic acid and then into glucose—the Cori cycle. The myocardial tissue can use lactate as an energy source, which is also converted to pyruvate due to the action of LDH. Pyruvate is further converted to acetyl-coenzyme A, which enters mitochondria and is metabolized in the Krebs cycle [68,69,70]. However, this pathway requires an oxygen supply and does not function in anaerobic conditions. In conditions of prolonged/excessive physical activity, there is an increased production and accumulation of lactate. An imbalance between the demand for oxygen by cardiomyocytes and the ability to deliver it through the coronary arteries leads to short-term transient ischemia and the transition of the myocardium to the anaerobic process. In such conditions, the myocardium ceases to utilize lactate and produces significant amounts of lactate itself. The accumulation of lactic acid in cardiomyocytes leads to acidification of the intracellular environment, which activates proteolytic enzymes and caspases (apoptotic enzymes), which break down sarcomeric proteins, including troponins, into smaller fragments, which will allow them to pass through the intact cell membrane [71,72]. At the same time, during ischemia, along with the proteolysis of troponins, proteolysis of cell membrane proteins occurs, which leads to increased permeability of the biomembrane and additionally promotes the release of troponins from the cardiomyocyte.

### 2.5. Increased Permeability of Cardiomyocyte Cell Membranes

Two main mechanisms are proposed: one of the mechanisms may be associated with proteolytic damage to cell membranes during ischemia. The second mechanism for increasing the permeability of biological membranes of cardiomyocytes and the release of troponins may be associated with stretching. A relationship has been established between the increased load on the myocardium and the release of troponins. Troponins are released, predicting adverse cardiac events, in the same way that natriuretic peptides (heart hormones) are released in chronic heart failure due to myocardial stretching [73]. It is believed that integrins play one of the most critical roles in this process. Integrins are transmembrane glycoprotein receptors that mediate communication between the intracellular and extracellular spaces. It was found that integrins, together with signaling molecules, stimulate myocardial distension. Hessel et al. used a special peptide to activate integrin and discovered a significant increase in troponin I in comparison to the control. The authors excluded ischemic and necrotic changes based on normal concentrations of lactate dehydrogenase, lactate, and the microscopic data [74]. It has been shown that the increase in troponins accompanies an increase in preload in the absence of ischemia. Feng et al. (2001) experimentally found that an increase in preload is accompanied by the activation of the endogenous intracellular enzyme calpain, which causes the proteolysis of troponin I into fragments with their further release from the myocardium. The preload was increased by inflating a special balloon inserted into the rat’s left ventricle; normal lactate concentrations confirmed the absence of ischemia. The administration of calpeptin, a calpain-specific inhibitor, and the elimination of preload reduced troponin I breakdown, its release from the myocardium, and improved left ventricular function. The data obtained indicate the possibility of an increase in troponins in the absence of myocardial ischemia; in this case, as the authors suggest, the mechanical stress of the myocardium led to the degradation of troponin and an increase in membrane permeability, which facilitated the release of troponin I into the blood [75]. Researchers note the possible important role of this mechanism in the pathogenesis and a possible focus for targeted therapy of heart failure in the presence of heart disease—more specifically, the valve leaflet insufficiency [75,76].

### 2.6. Small-Scale Myocardial Cell Necrosis

MRI with gadolinium contrast showed no foci of necrotic or sclerotic changes in healthy athletes [77]. Despite this, some researchers believe that some athletes develop small-scale (subclinical) necrosis. An argument in favor of the death of myocardial cells during high-intensity physical exertion is that the concentration of cardiac troponins under heavy loads, particularly after a marathon run, can increase by 8–10 times [78]. Furthermore, the gadolinium-enhanced MRI technique still has insufficient sensitivity compared to laboratory diagnostics. 

Scherr et al. (2011) investigated troponin T by a highly sensitive method (Roche Diagnostics) dynamically in the blood of marathon runners. Immediately after the finish, troponin T levels were 0.031 ng/mL, which was about ten times higher than normal, and the concentrations returned to normal after 72 h. Approximately similar tendencies were shown by cardiac fatty acid-binding protein and N-terminal brain natriuretic peptide (NT-proBNP) [79]. Thus, the emergence of highly sensitive methods for the detection of troponins creates the need to revise and standardize the permissible physical activity for the safety and subsequent preservation of the health of athletes, which needs further research and clarification. 

Severe psychoemotional stresses, imbalances in the neurohormonal system, and subclinical inflammation of the heart tissues (myocarditis, etc.) can be other presumptive causes of minor necrosis [80,81]. Lazzarino et al. (2013) found an association between elevated stress hormone (cortisol) concentration and troponin T detection by the highly sensitive method (Roche Diagnostics) in healthy study participants (odds ratio (OR): 3.98; 95% confidence interval (CI): 1.60 to 9.92; *p* = 0.003). The authors emphasize the need for further research to clarify the role of psychoemotional stress in the pathophysiology of cardiomyocyte damage [82,83]. Stress can be a trigger of myocardial infarction and occur in people who have already had a heart attack (worries, a feeling of fear of death). However, difficulties arise in the differential diagnosis of AMI because an elevated level of a highly sensitive troponin I against the backdrop of transitory ischemia/angina pectoris and stress might lead to the overdiagnosis of AMI. Table 1 summarizes the pathways hypothesized for the release of cardiac troponin molecules from cardiomyocytes.

## 3. Features of the Troponin Circulation in the Bloodstream

It is essential to understand that troponins circulate in the blood in the form of a heterogeneous pool: free (single) troponin T and I molecules, combined (binary and triple) troponin complexes, fragments of proteolytic cleavage of troponins, as well as their oxidized, phosphorylated and glycosylated derivatives. The half-life of troponins released into the bloodstream, according to some data, averages 1–2 h [87].

Released into the bloodstream, cardiac troponins and/or their fragments, unlike many biologically active compounds (hormones), do not perform any regulatory functions but circulate for some time, serving as specific biomarkers of myocardial damage, and then are eliminated. Cells of the reticuloendothelial system (mainly macrophages of the spleen) play a significant role in purifying blood from troponin molecules [88,89]. Presumably, increased cleavage of cardiac troponins occurs in conditions of splenomegaly (hypersplenism), by analogy with the accelerated disintegration of blood corpuscles. 

Furthermore, the previously described cleavage of troponins by proteases, which happens in cardiomyocytes under normal conditions but becomes more intense in borderline or pathological conditions, facilitates their release into the blood and subsequent removal.

Along with the intracellular cleavage of cardiac troponins (in cardiomyocytes, macrophages), proteolysis of these molecules occurs directly in the vascular bed. It has recently been found that troponins can be specifically cleaved by the enzyme thrombin directly in the bloodstream [24]. Moreover, the introduction of a specific thrombin inhibitor did not lead to the cleavage of troponin T [24]. It should be assumed that many other proteolytic enzymes, including other enzymes of the hemostatic system, participate in the extracellular destruction of troponins, which requires further investigation. The hemostatic system is a delicate balance of coagulation and anticoagulation mechanisms under normal conditions, pathology, and/or drug intervention tips the balance to one side, resulting in an indirect change in the heterogeneous fraction of circulating troponin protein fragments. Furthermore, this can subsequently affect the result of the analysis.

Katrukha et al. used Western blotting with monoclonal antibodies specific for distinct troponin I epitopes to measure serum samples from patients with acute myocardial infarction (AMI) within 1–36 h of the beginning of chest pain. In addition to free (intact) troponin I, the researchers found 11 more fragments of this molecule with different molecular weights and stability in the bloodstream. When studying the stability of molecules, it is noted that the least stable regions of the troponin I molecule are the N- and C-terminal regions. At the same time, the central fragments are more stable and acquire high resistance to proteases due to their binding to troponin C. The authors draw attention to the extreme importance of studying the processes of fragmentation of troponin molecules and their stability under various conditions for the development and improvement of methods for determining troponins [90]. 

Zahran et al. examined serum/plasma samples from 29 patients with different types of infarction for proteolytic degradation using several ELISA kits designed to determine the N-terminal, nuclear, and C-terminal fragments of troponin I. Patients with type 1 AMI (with acute atherothrombosis) and ST-segment elevation had the highest degree of proteolytic cleavage of troponin I molecules; patients without ST elevation, as well as patients with type 2 AMI (with an imbalance in demand and oxygen delivery), had the lowest degree of proteolytic cleavage. The lowest degree of proteolytic degradation was in patients after percutaneous coronary intervention. As a result, the authors concluded that the degree of proteolytic degradation is a better indicator of ischemia and AMI than the total level of serum troponin. It was discovered that the degree of troponin proteolytic cleavage corresponds to the severity and prognosis of AMI more than the overall concentration of troponin I in blood serum. In addition, this work indicates that it is possible to assess the quality of treatment based on determining the degree of fragmentation of troponin I [26]. 

Studies on troponin degradation processes explain why commercial antibodies to unstable (rapidly degraded) epitopes at the N- and C-termini of troponin I have a poor correlation with clinical data and patient prognosis when compared to test systems that use antibodies to central (more stable) regions of the molecule. Further research on troponin intra- and extracellular cleavage is needed to improve and standardize troponin immunoassays. In our opinion, the most important goals of such studies should be:

1. conducting clinical studies for a comparative assessment of the diagnostic effectiveness of already existing highly sensitive immunoassays;
2. searching in blood and other biological fluids for fragments of troponins, which are released first of all during ischemia or AMI;
3. developing antibodies to these (the early released) troponin fragments, which will allow earlier diagnosis of AMI;
4. studying the lifespan/circulation of troponin fragments in the bloodstream and biological fluids to optimize laboratory diagnostics
5. comparative studies will probably reveal the most specific troponin fragments specifically for AMI, but not for other conditions (exercise, myocarditis, pulmonary embolism, etc.), which may be accompanied by an increase in troponins when using currently existing test systems and significantly complicate differential diagnosis.

## 4. Elimination of Cardiac Troponins from the Blood

The exact mechanisms of eliminating the cTnI and cTnT protein molecules from the bloodstream and the factors that influence the elimination of troponins are not entirely known. The elimination of cTnI and cTnT molecules from the blood is thought to occur in three ways: (1) capture of protein molecules cTnI and cTnT by cells of the reticuloendothelial system and intracellular cleavage [91,92,93]; (2) cleavage of cTnI and cTnT molecules by proteolytic enzymes directly in the bloodstream [23,24]; (3) elimination of cTnI and cTnT molecules through the blood–tissue filters/barriers (glomerular and blood–salivary) into other biological fluids (urine and oral fluid) [28,29,30,94,95]. Some of the elimination mechanisms may be closely related to each other. For example, the cleavage of intact cTnI and cTnT molecules into smaller molecular fragments will enhance the elimination of these proteins through glomerular filtration. It is critical to understand that factors influencing these elimination pathways for cTnI and cTnT molecules will affect serum cTnI and cTnT levels. As a result, if the elimination of cTnI and cTnT molecules decreases, they accumulate in the bloodstream, and the concentration of cTnI and cTnT in the blood serum may increase; if elimination increases, the concentration of cTnI and cTnT in the blood hypothetically may decrease. Given the potential impact of elimination pathways on serum levels of cardiac troponins, it is critical to focus on studying these mechanisms and determining the specific contribution of each mechanism, which is likely to explain some of the false-positive or false-negative results that occur in clinical practice. 

Renal filtration is one of the most controversial mechanisms for eliminating cTnI and cTnT molecules from the bloodstream [91,92]. Due to the small size (molecular weight) of troponins and their fragments, eliminating these molecules from the bloodstream is carried out through the renal filter using glomerular filtration. Molecules of cardiac troponins have been detected in urine in various recent studies [20,37,92], which can be considered direct evidence of the presence of this troponin elimination pathway. Along with these data, it has been shown that inhibition of filtration processes through the glomerular filtration due to chronic renal failure (CRF) leads to an increase in the bloodstream troponin levels without any damage to cardiomyocytes [95,96,97].

Despite this, due to a lack of direct proof, not all scientists recognize renal filtration as the principal mechanism for eliminating cardiac troponins from the blood [98,99,100,101]. A recent study by Croatian scientists led by Pervan et al. (2017) using a highly sensitive immunoassay (Abbott Architect) revealed troponin I in all examined patients. In addition, it was shown that the level of a highly sensitive troponin I was higher in the urine of hypertensive patients compared to normotensive patients (*p* = 0.0451); this, according to the authors, can be used in clinical practice for the diagnosis and monitoring of arterial hypertension [92]. 

In the normal range of blood pressure in healthy individuals, the glomerular filtration is not dependent on blood pressure levels because of the Bayliss effect (myogenic mechanism). However, under conditions of strongly pronounced changes in blood pressure, the glomerular filtration rate (GFR) will change: a significant increase in blood pressure leads to an increase in the (GFR), which is a calculated value based on the concentration of the endogenous metabolite creatinine. Considering that an increase in blood pressure increases the troponin concentration in the urine, this validates the direct dependency of serum concentrations of cardiac troponins on GFR. An increase in serum troponin values accompanies a decrease in GFR. This is confirmed by the data of a large study, which involved 2464 patients with chronic renal failure. Participants with a GFR < 30 mL/min/1.73 m^2^ had approximately three times larger highly sensitive troponin T values than those with a GFR greater than 60 mL/min [95]. GFR suppression is observed in chronic renal failure and various polyetiological hypotensive conditions (taking drugs, shock, etc.). As a result of the pronounced decrease in blood pressure observed in cardiogenic shock, which frequently occurs with large-focal myocardial infarctions, troponin levels in the blood and the duration of their circulation in higher concentrations increase, which is also considered a prognostically unfavorable sign.

Understanding the characteristics of troponin elimination is critical when utilizing rapid algorithms for the diagnosis or exclusion of AMI, as demonstrated in the study by Kavsak et al. (2018) [102]. Highly sensitive troponin I (Abbot) and T (Roche) concentrations were negatively correlated with GFR: the lower the GFR, the higher the troponin levels. At the same time, the authors observed that the current troponin threshold values (99th percentile) for excluding AMI are only appropriate for individuals with a GFR of 90 mL/min/1.73 m^2^. Moreover, this suggests that the increased values of cardiac troponins in patients with lower GFR may be due to a cause that has nothing to do with ischemic damage to cardiomyocytes, leading to AMI overdiagnosis and unnecessary costs for unnecessary therapeutic and diagnostic manipulations. It is evident that for the most optimal use of highly sensitive analyzes, physicians must necessarily consider GFR [95]. It should be noted that to date, the current clinical guidelines do not consider the renal mechanism of elimination of cardiac troponins and additional studies are needed to clarify this mechanism. Additional research is needed to adjust the troponin thresholds in relation to different GFR values. It is possible to develop separate diagnostic algorithms for excluding AMI for patients with chronic renal failure. 

It has been reported that troponin fragments are removed across the blood-salivary barrier. However, the specific process has not been determined. This is most likely accomplished through ultrafiltration of small fragments of troponin proteolytic degradation. In the case of AMI, an increase in troponin concentration in the circulation leads to an increase in troponin concentration in the oral fluid [103]. Another study, conducted by Bunin et al. (2017), revealed that troponin I concentrations in the blood serum and saliva of patients with ischemic heart disease (IHD) are positively related to the stage of the disease. Thus, in the salivary fluid of older adults without cardiovascular pathology, the average amount of troponin I was 0.67 ng/mL. In patients with stages 1 and 2 of the development of coronary artery disease, the mean concentration of troponin I significantly increased by 2 and 3.4 times, respectively (up to 1.37 and 2.28 ng/mL). In individuals with stage 3 IHD, the mean troponin I concentration increased 5.1 times (up to 3.42 ng/mL) [104]. Such information can be used for non-invasive diagnosis and monitoring of coronary artery disease and myocardial infarction.

Several studies have identified cardiac biomarkers, including troponins, in cerebral fluid and pericardial fluid during postmortem examination, which may help estimate the degree of the myocardial injury. The entry of troponins into the pericardial fluid is due to the proximity of the location. Higher concentrations are observed in transmural myocardial infarction. The presence of troponins in the cerebrospinal fluid indicates their ability to cross the blood–brain barrier [31,32]. 

Stefanovic et al. found cardiac troponin T in the amniotic (amniotic) fluid, while its concentration was positively correlated with erythropoietin levels (*r* = 0.526; *p* = 0.003). Erythropoietin is produced in the cells of the juxtaglomerular apparatus of the kidneys in response to hypoxia. The authors believe that troponin detection in amniotic fluid should be performed in pathological pregnancy to diagnose fetal hypoxia, myocardial damage, and malformations [34].

Table 2 summarizes the above mechanisms for eliminating cTnI and cTnT molecules from the bloodstream. 

## 5. Circadian Aspects on the Cardiac Troponins Concentration Fluctuations

Circadian concentration variation is typical for most analytes [65,107,108,109]. The most severe daily fluctuations are seen in the levels of hormonal parameters and metabolic components which these hormones affect. Several studies have shown the presence of circadian fluctuations in troponin concentration, based on which it was believed that they should be considered when setting the values of the 99th percentile, and the delta increase in troponin concentration in patients admitted to the emergency department with suspected acute myocardial infarction [64]. This is due to the fact that early algorithms used to diagnose myocardial ischemia are based on relatively low troponin concentrations [5,6,110,111], and even slight variations due to circadian aspects can lead to false-positive or false-negative interpretations of the analysis results. 

Recently, it was shown that troponin T, determined by a highly sensitive method (Roche Diagnostics), has a significant daily fluctuation in concentration: the maximum level of highly sensitive troponin T is detected in the morning hours (8:00), then a decrease is observed and reaches its minimum value by about 20:00, after which a gradual increase in concentration begins again, which again reaches a maximum in the morning. The average values in the morning and evening were 16.2 ng/L and 12.1 ng/L. The authors believe that the circadian rhythm of troponin release (according to highly sensitive methods) should also be considered for screening purposes [111]. 

In some cases, the circadian features of troponins can affect the diagnostic accuracy for AMI. van der Linden et al. (2016) estimated the daily fluctuations in hs-TnT and hs-TnI concentrations in an elderly patient with severe chronic renal failure (GFR = 14 mL/min). Their serum cardiac troponin T concentration had been chronically elevated for many years with the absence of acute coronary events, highlighting the role of the kidney in troponin elimination. Changes in troponin T levels during the day were in line with the previous study, with peak concentrations in the morning and a gradual decline in the evening. During the day, the maximum fluctuations in troponin T concentrations were 50.9 ng/L and up to 20 ng/mL within one hour. Several times a day, one-hour delta-changes in troponin T levels exceeded delta values in the developed one-hour and three-hour algorithms for diagnosing AMI. In other words, in this case, circadian fluctuations in troponin T concentrations mimicked the kinetics of troponin T characteristic of AMI, which could lead to overdiagnosis [112]. 

Few works are devoted to circadian features, and there is a need for further research, especially for modern highly and ultrasensitive test systems. When significant influences are found, the diagnostic algorithms for AMI should reflect the circadian aspects [64]. When it is confirmed that morning troponin levels are greater than evening troponin levels, higher cut-off values (99th percentile) should be utilized for patients admitted in the morning with suspected AMI. So, for example, at the moment, circadian features have been studied in detail and are successfully used for several hormonal parameters in endocrinology. 

The daily concentration variability characteristic of cardiac troponin T corresponds to the circadian organization of the cardiovascular system and the hemostatic system. It has been shown that the heart rate, blood pressure, vascular resistance, prothrombotic tendency, platelet aggregation ability, activity of the renin-angiotensin-aldosterone system, activity of the sympathoadrenal system, and levels of catecholamines and cortisol increase in the morning hours, which have evolved and are necessary for normal functioning in the period of wakefulness. However, this has important implications for the pathophysiology of cardiovascular diseases: the frequency of cardiovascular accidents is significantly higher in the morning hours [113,114,115,116,117,118]. The maximum number of cases of AMI occurs in the morning–afternoon time interval (8:00–12:00). It was discovered that patients hospitalized in the morning have a larger AMI focus than those admitted in the evening and night, which is associated with increased sympathoadrenal axis activity [119]. It should be noted that to date, current clinical guidelines do not contain information on the effect of circadian rhythms of cardiac troponins and additional clinical studies are needed to validate them.

## 6. Conclusions

Taking everything into account, it is possible to conclude that the concentration of cardiac troponins at a specific point when drawing blood from a patient is affected by a variety of metabolic factors (release, circulation, cleavage and removal, circadian features). This must be understood to use fast algorithms to diagnose and exclude acute myocardial infarction, based on small concentration rises (several ng/L) during the first hours. It should be understood that some of the above factors can lead to a distortion of the result; therefore, additional fundamental studies of the metabolic characteristics of cardiac troponins are needed to improve the diagnostic process. According to the literature review, the following significant influencing features that can lead to diagnostic errors should be considered: troponin release during stress, transient ischemia, and apoptosis of cardiomyocytes; impaired renal elimination, which can overestimate troponin concentrations in the absence of cardiomyocyte damage; and circadian aspects. Further research of troponin circulation in the bloodstream and troponin lifespan is required to develop novel diagnostic systems (for example, the creation of antibodies to those troponin fragments that appear first in the blood during myocardial infarction and have the longest lifespan under certain conditions). Determining troponins in other biological fluids (pericardial, cerebrospinal, amniotic, urine, and oral fluid) opens up new questions and perspectives.

## Figures and Tables

**Table 1:** Mechanisms of cell release/increasing troponin cTnI and cTnT concentrations.

Mechanisms of Release of cTnI and cTnT Molecules from Cardiomyocytes	Additional Comments	Sources
Reversible (subclinical) and irreversible damage (necrosis) of cardiomyocytes	When cells are damaged, the cytoplasm (sarcoplasm) contents are released into the extracellular space, including troponin proteins. Troponin elevation correlates with the degree of damage to cardiomyocytes.	[2,5]
Cardiomyocyte apoptosis	Myocardial cell apoptosis occurs both during short-term ischemia due to caspase enzyme activation and through non-ischemic causes (with stretching of the myocardium, increased adrenergic stimulation through beta-adrenergic receptors)	[55,56,57,58,60]
Myocardial regeneration processes (?)	Several studies have found evidence of minor cardiomyocyte regeneration. Cell regeneration and renewal are accompanied by releasing a limited amount of cTnI and cTnI molecules into the surrounding environment. According to the researchers, this process explains the presence of troponins in the serum of all healthy people	[39,40,41,42]
Increased permeability of the cardiomyocyte cell membrane	The permeability of cardiomyocyte cell membranes increases with stretching and cardiac ischemia due to the activation of proteolytic enzymes, which in turn damage the cell membranes, allowing troponin molecules to be released outside the cell	[62,67,74]
Processes of intracellular cleavage of protein molecules by proteolytic enzymes	Troponins are cleaved into smaller fragments within the cell by various proteolytic enzymes, including caspases and calpain. Smaller pieces are generated due to proteolytic cleavage, which can probably pass through the intact membrane of the cardiomyocyte. These enzymes can be triggered after myocardial ischemia and an increase in myocardial load. Thus, this process underlies the increase in troponin levels associated with ischemic myocardial diseases (ischemic heart disease, myocardial infarction) and various physiological or pathological conditions characterized by an increase in the load on the myocardium. Furthermore, the methods of intracellular proteolytic cleavage of the cTnI and cTnI molecules are highly likely to be associated with an increase in membrane permeability caused by damage to the cardiomyocyte’s cell membrane by the same proteolytic enzymes.	[74,75,76]
Formation and release of membrane vesicles (vesicular transport)	Using electron microscopy methods on hepatocytes and cardiomyocytes (in vitro), it was discovered that during the initial stages of ischemia (before the development of necrosis in cardiomyocytes), vesicles are formed on the surface of the cell membrane, within which cytoplasmic proteins can be localized, including the cytoplasmic fraction of troponins. The release of troponins into the extracellular space hypothetically occurs when these vesicles rupture.	[66]
Re-expression of cardiac troponin molecules in striated muscle in skeletal myopathies and troponin release into the bloodstream due to subsequent damage to muscle fibers (?)	Several research groups have described the re-expression of cardiac troponins in damaged skeletal muscle fibers [84,85]. However, this mechanism is considered controversial. Other researchers suggest that the reason for the increase in cardiac troponins in patients with skeletal myopathies is false-positive (cross) reactions between diagnostic (cTnI and cTnT) antibodies and skeletal troponin isoforms [86].	[84,85,86]

**Table 2:** Mechanisms of elimination of cardiac troponins from the blood.

Mechanisms of Cardiac Troponin Elimination	Comments	Sources
Cleavage of cTnI and cTnT molecules by the cells of the reticuloendothelial system	Protein molecules are captured by the cells of the reticuloendothelial system (tissue macrophages, Kupffer cells, etc.), where they undergo proteolytic destruction. A similar mechanism is relatively well described for some cardiac markers (creatine kinase, lactate dehydrogenase, aspartate aminotransferase) and is probably also a characteristic of cTnI and cTnT.	[93,94,105]
Cleavage of cTnI and cTnT molecules by proteolytic enzymes directly in the bloodstream	All protein molecules, including cTnI and cTnT, are sensitive to the action of proteolytic enzymes. Some enzymes (caspase, calpain, and thrombin) have been found to cause proteolytic cleavage of cTnI and cTnT molecules into molecular fragments, according to some studies.	[23,24,25,26,90,106]
Elimination of cTnI and cTnT molecules through the glomerular and blood-salivary barriers into urine and oral fluid	Clinical studies have demonstrated the existence of this mechanism and are considered to have a potential diagnostic value. There was also a link between serum and salivary troponin levels, according to the researchers. This elimination mechanism is closely related to the mechanisms of troponin cleavage. As a result of proteolytic cleavage, small molecular fragments can be formed, which can much more easily (and, accordingly, in large quantities) pass through the renal and blood–salivary barriers. Clarification of these mechanisms is very likely to increase the diagnostic value of cTnI and cTnT in these biological fluids.	[20,28,29,30,92,106]

## Data Availability

Not applicable.
